# Oxidative Modification of LDL by Various Physicochemical Techniques: Its Probable Role in Diabetes Coupled with CVDs

**DOI:** 10.1155/2018/7390612

**Published:** 2018-11-18

**Authors:** Sultan Alouffi, Mohammad Faisal, Abdulrahman A. Alatar, Saheem Ahmad

**Affiliations:** ^1^Department of Clinical Laboratory Sciences, College of Applied Medical Sciences, University of Hail, Saudi Arabia; ^2^Department of Botany & Microbiology, College of Science, King Saud University, P.O. Box 2455, Riyadh 11451, Saudi Arabia; ^3^Department of Biosciences, Integral University, Lucknow, Uttar Pradesh-226026, India; ^4^IIRC-1, Laboratory of Glycation Biology and Metabolic Disorder, Integral University, Lucknow, Uttar Pradesh-226026, India

## Abstract

**Background:**

Pro- and antiatherogenic properties of oxidised low density lipoprotein (Ox-LDL) are responsible for different chronic diseases including diabetes and cardiovascular diseases (CVD). The constant attack on the body from oxidative stress makes the quantification of various oxidation products necessary. In this study, the oxidative stress causing the structural and chemical changes occurring in the LDL molecule is comprehensively done. Moreover, the prevalence of the autoantibodies against the oxidised LDL is also determined.

**Methods:**

Our study made an attempt to see the effect of Ox-LDL as an enhancer of type 2 diabetes mellitus (T2DM) coupled with CVD. Primarily, we detected the oxidation of LDL with different concentration of Fenton reaction. The biochemical parameters were assessed for the changes occurring in the LDL molecule. In a clinical set up, 20 sera samples were taken from patients who are healthy, 30 from those with diabetes, 20 from those with CVD, and 30 from diabetes with CVD patients.

**Results:**

In biochemical assays there were markedly increased TBARS, carbonyl, and HMF content in Ox-LDL as compared to native LDL. The prevalence of autoantibodies against the T2DM was recorded to be 36%, while for CVD it was recorded to be 29%. However, it was found that 50% of the sera samples showed autoantibodies against oxidized LDL in the sera of T2DM with CVD complications as compared to the native analogue.

**Conclusion:**

There is significant change in the LDL molecule as revealed by various physicochemical analysis. The change in the LDL macromolecule as a result of oxidation triggered the development of the autoantibodies against it.

## 1. Introduction

ROS have long been involved in the oxidative damage imposed on fatty acids, DNA, and proteins as well as other cellular components resulting in carcinogenesis, neurodegeneration, atherosclerosis, diabetes, and ageing [1–3]. When ROS overpower the cellular antioxidant defense system either through an increase in free radicals or decrease in the cellular antioxidants, it results in the oxidative burden in the cells producing oxidative burst. The production of the highly reactive ROS takes place due to stepwise reduction of molecular oxygen (O_2_) by high energy exposure or electron-transfer reaction. Cellular ROS are generated within an organism as in the process of mitochondrial oxidative phosphorylation, or they may arise exogenously, such as by interactions with xenobiotic compounds. It includes nonradical molecules like hydrogen peroxide (H_2_O_2_), singlet oxygen (_1_O_2_), as well as free radicals such as superoxide anion (O_2_∙^−^), and hydroxyl radical (^*∙*^OH) [4]. The most highly reactive species ^*∙*^OH is formed in a Fenton reaction, in which H_2_O_2_ reacts with metal ions (Fe^+2^ or Cu^+^), which act as catalyst, often bound in complex with different proteins such as ferritin (an intracellular protein that stores iron) and ceruplasmin or other molecules [5]. Under stress conditions, an excess of O_2_∙^−^ releases free iron from ferritin and the released free iron takes part in Fenton reaction to form OH^*∙*^ [6]. These ^*∙*^OH formed can damage DNA, lipids, and proteins as well.

Among all the proteins in humans, LDL is a metabolic endpoint for apolipoprotein B (apoB) containing lipoproteins and circulates within the vascular compartment, including the subendothelial space, until removed by high-affinity apoB receptor-mediated endocytosis. Exposure of LDL to ROS chemically damages LDL, creating lipid peroxides and ultimately protein adducts of apoB. Oxidative modification of apoB alters its ligand properties and marks it for removal by scavenger receptors. Ox-LDL directly delivers various lipid-oxides and hydroperoxides to target cells. These compounds variably act as cytotoxins, monocyte chemoattractants, and stimulators of cholesterol ester accumulation by macrophages and inhibitors of macrophage movement [7]. In other word, when the LDL cholesterol particles in our body react with free radicals, the oxidation of LDL occurs. The Ox-LDL itself then becomes more reactive with the surrounding tissues, which can cause tissue damage. The level of Ox-LDL is increased by diet that is high in transfats, smoking, having poorly controlled diabetes, and being diagnosed with metabolic syndrome. It is considered an early marker of oxidative stress and lipid peroxidation. So the need of studying and understanding its harmful impact on the human body becomes necessary. The relation between Ox-LDL and T2DM has been successfully established primarily in 2016 [8]. In the present study, oxidation of LDL was analyzed through the UV-Vis spectroscopy, fluorescence spectroscopy analysis, and various biochemical assays. Moreover, the autoantibodies which might have generated against native and Ox-LDL in the sera of type 2 diabetes with CVD were also probed.

## 2. Materials and Methods

*Ethical Clearance*. The work was ethically approved by ethics committee of the Integral University with an approval no. IEC/IIMSR/2017/39.

### 2.1. Materials

Ferric chloride, hydrogen peroxide, phosphate buffer saline (PBS), bicarbonate buffer, thiobarbituric acid (TBA), and trichloroacetic acid (TCA) were obtained from sigma chemical company. Nitroblue tetrazolium chloride (Hi Media), Dialysis membrane (Hi Media), BCA estimation Kit (G Biosciences), and 96 well plates (Nunc) were obtained. The other reagents used were tri-sodium citrate buffer, 2,4-dinitrophenylhydrazine (DNPH), guanidine hydrochloride and 9,10-phenanthrenequinone, Tween-20, anti-rabbit IgG, dialysis tubing, and p-nitrophenyl phosphate. All other chemicals used in this study were of analytical grade available.

### 2.2. Isolation of LDL from Human Blood

Blood sample (5 ml) was taken in an EDTA coated vial so as to prevent clotting. It was centrifuged at 2000 rpm for 15 minutes at 25°C and the plasma (supernatant) was isolated through pipette. The plasma was stored at −20°C. To isolate LDL, one volume of the plasma was added to the seven volume of tri-sodium citrate along with heparin buffer and vortexed for uniform mixing. Then it was left at room temperature for 10 minutes. The mixture was centrifuged at 1500 rpm for 10 minutes at 25°C. The supernatant was discarded and the pellet was dissolved in 1 volume of PBS buffer (pH 7.4). LDL concentration was estimated before keeping at 4°C [9].

### 2.3. BCA Protein Estimation

The BCA protein assay is used for quantification of total LDL in a sample. The principle of this method is that proteins can reduce Cu^+2^ to Cu^+1^ in an alkaline solution and result in a purple colour formation by bicinchoninic acid. The reduction of copper is mainly caused by four amino acid residues including cysteine, tyrosine, and tryptophan that are present in protein molecules [10]. 2 mg/ml bovine serum albumin (BSA) stock was prepared in distilled water and taken as standard. Seven test tubes were then labelled and 500 *µ*l double distilled water was added to each tube. 500 *µ*l from the stock solution was added to the first tube and serial dilution was done in rest of the tubes. Another set of eight test tubes were taken and to each tube; 1 ml of BCA reagent (50:1 ratio of BCA and copper solution) was added. To the first tube, 50 *µ*l stock solution was added and 50 *µ*l solution from the test tube 1 of first set was added in the second tube of another set and so on. For LDL, TCA precipitation was done to degrade the lipid part from LDL leaving only the protein part, by dissolving 100 *µ*l LDL in 1 ml TCA and vortexing. The LDL-TCA mixture was centrifuged at 10,000 rpm for 15 minutes. Supernatant was discarded and the pellet was dissolved in 100 *µ*l PBS. Then 50 *µ*l was taken from that and 1 ml BCA solution was added to it. The tubes of the second set and the tube containing LDL and BCA solution were incubated at 37°C for 10 minutes. The absorbance readings were taken at 562 nm [9].

### 2.4. Oxidation of LDL by Fenton Reaction

60 *µ*g of LDL was taken for 1 ml reaction. 2 tubes were labelled as test and native. The tube labelled as “test” consisted of 60 *µ*g of LDL, 16.6 *µ*M of FeCl_3_, and 500 *µ*M of H_2_O_2_ from and rest of the volume had PBS (0.1 M, pH 7.4). In the tube labelled as “native” only LDL and PBS were taken, excluding Fenton reagents. Both tubes were covered with black sheet and the reaction is carried out in the dark room. The samples were incubated at 37°C for 1 hour. After incubation, the absorbance was taken at 282 nm on UV-Vis spectroscopy (Eppendorf).

### 2.5. UV-Vis Spectroscopy

The absorption profile of LDL sample was recorded on a spectrometer (Eppendorf) in quartz cuvette of 1 cm path length wavelength ranging from 240-800 nm. An individual aliquot of the native and oxidized LDL was analyzed for absorbance at 282 nm [11].

### 2.6. Dialysis of Ox-LDL

This method was used for the removal of free sugars from the glycated proteins via dialysis against PBS (ph 7.4). Special dialysis tubes were utilised and sterilized after being boiled for at least 30 minutes in a solution composed of 10 mM PBS/1 mM EDTA. One end of the tube was tied then the protein samples were poured into the tube after which the other end of the tube was sealed. The dialysis tubing was transferred into a beaker containing 1 litre of distilled PBS. Dialysis was conducted by stirring the samples at room temperature. The water was exchanged at 4 hours' time interval and then finally kept for overnight stirring. Finally, the samples were transferred into clean tubes and stored at -20°C until further use.

### 2.7. Nitroblue Tetrazolium (NBT) Reduction Assay

Superoxide radicals in oxidized LDL sample was determined by NBT reduction test. The LDL samples (20 *µ*l) were mixed with 180 *µ*l of 0.25 M nitroblue tetrazolium (NBT) and incubated at 37°C for 20 minutes. The purple colour formation (absorbance) was read at 525 nm against sodium carbonate–bicarbonate (pH 10.8) and contents of LDL oxidized product were measured [12].

### 2.8. Measurement of Hydroxyl Radical in Oxidized Sample by TBARS

Detection of hydroxyl radicals was carried out according to the following protocol. 500 *µ*l of LDL samples was taken and the degradation was measured by adding 1 ml of 2.8% (w/v) trichloroacetic acid + 1 ml of 1% (w/v) thiobarbituric acid, followed by heating at 100°C for 10 min. After cooling, the absorbance was read at 532 nm. The reaction mixture containing phosphate buffer saline (pH 7.4) was used as the blank [13].

### 2.9. Measurement of Hydroxy Methyl Furfural (HMF)

100 *µ*l of the LDL sample was mixed with 500 *µ*l oxalic acid (1 M), followed by boiling at 100°C for 1 hour. It was then left at room temperature for 10 minutes to bring the temperature down. After cooling, 500 *µ*l of 40% TCA was added and the mixture was centrifuged at 8000 rpm for 10 minutes at 4°C. To the supernatant, 500 *µ*l TBA (0.05 M) was added and incubated at 40°C for half an hour. The absorbance readings were taken at 443 nm against TBA, which was used as a blank [13].

### 2.10. Measurement of Carbonyl Content

To the 100 *µ*l sample, 400 *µ*l of 10 mM DNPH was dissolved and kept at room temperature for 60 minutes. 500 ul TCA (20%) was added and left on ice for 5 minutes. The reaction mixture was centrifuged at 10,000 rpm for 10 minutes at 4°C. The supernatant was discarded and the pellet was washed three times using 500 *µ*l of 1:1 ethanol and ethyl acetate. To the final pellet, 250 *µ*l of 6M guanidine hydrochloride was dissolved and the absorbance was read at 370 nm. The blank used was guanidine hydrochloride [14].

### 2.11. Estimation of Free Arginine

1 ml of the sample containing arginine was mixed with 3 ml of 9,10-phenanthrenequinone (150 *µ*M). 0.5 ml of 2N NaOH was then added and incubated at 30°C for 60 minutes. After incubation, equal volume of 1.2M HCl was mixed and fluorescence was measured at 395 nm using an excitation wavelength of 312 nm [15].

### 2.12. Direct Binding ELISA

Direct binding ELISA was performed on polystyrene plates with slight modification. One hundred microtitres of 10 *µ*g/ml of LDL samples (in TBS, pH 7.4) was coated in microtitre wells and incubated for 2 hours at 37°C. The ELISA plate was then left overnight at 4°C. The samples were coated with half of the plate, and the rest half, which was devoid of antigen, served as control. The first two wells of the column 1st, 4th, 7^th^, and 10th were left as blank. The second day, all the wells of the plate were emptied and washed thrice with TBS-T to remove the unbound antigen. Unoccupied sites were then blocked with 100 *µ*l of 2.5% bovine serum albumin (BSA) and left for 4-5 hours at 4°C, followed by a single wash with TBS-T.100 *µ*l of the antibodies were coated in duplicate in all the wells, leaving blank, and incubated at 37°C for 2 hours and left overnight at 4°C. Next day, the wells were emptied and washed with TBS-T. Secondary antibody, i.e., anti-immunoglobulin alkaline phosphatase conjugate, was added to each well except blank. After 2 hours of incubation at 37°C, plate was washed three times with TBS-T followed by a single wash with distilled water. Then the substrate, i.e., paranitrophenyl phosphate was added in all the wells including blank and incubated for 45 minutes. The colour/absorbance was read at 415 nm on a microplate reader. The results were expressed as mean of Atest–Acontrol [11].

## 3. Results

### 3.1. Standard Curve for Protein Estimation by BCA Kit

The assayed samples of isolated LDL emerge dark purple color as compared to standard bovine serum albumin (BSA), which shows the presence of lipoproteins in the sample. Upon cooling at room temperature of assay, spectral reading was taken at 562 nm. With the help of spectral reading, the graph of concentration against absorbance for BSA was plotted and the concentration of isolated LDL was estimated to be 1.2 mg/ml (data not shown).

### 3.2. UV-Vis Spectroscopic Characterization of Modified LDL

**Figure 1:**
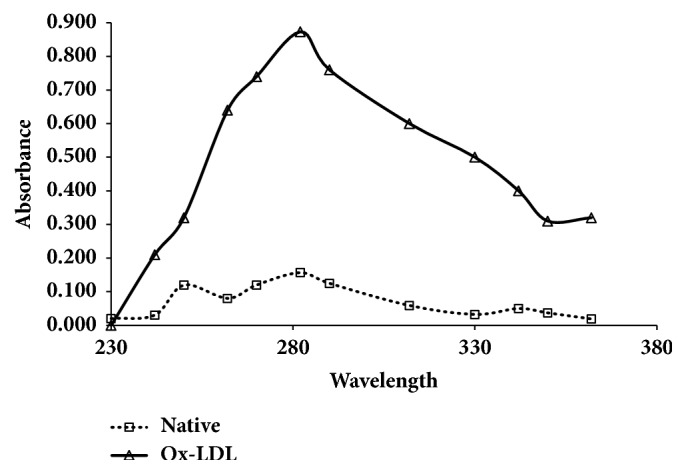
UV absorption spectra of native LDL and oxidised LDL (Ox-LDL) after 30 minute. The experiments were performed in triplicates.

The oxidized sample of LDL showed enhanced absorbance as compared to the native LDL. Hyperchromicity at 280 nm was recorded after 30 minutes of incubation of LDL to Fenton reaction (Figure 1). The hyperchromicity of modified sample at 280 nm was observed to be 82.65% (Table 1) which reflects the exposure of chromophoric aromatic amino acid residues due to the unfolding and fragmentation of protein upon oxidation.

### 3.3. Spectrophotometric Measurement of Superoxide Anion Radicals

**Figure 2:**
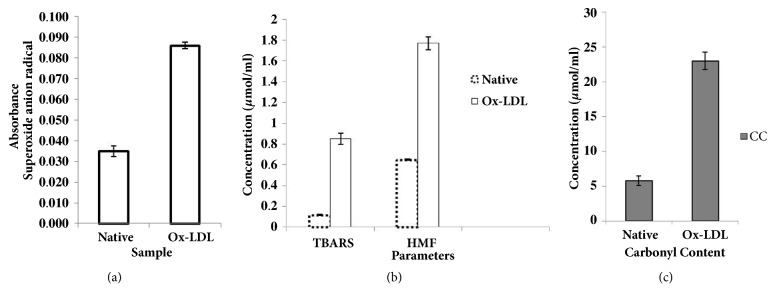
The biochemical changes in native and Ox-LDL: (a) superoxide (b) TBARS and HMF (c) and carbonyl content.

The nitroblue tetrazolium (NBT) test provides a simple assay for O_2_∙^−^ production. NBT is reduced by O_2_∙^−^ to a blue formazan product which is measured at 525 nm. The absorbance for Ox-LDL was higher (0.085) as compared to the native LDL (0.035) (Figure 2(a)). The percent increase in superoxide radical in the modified sample was 58.82% (Table 1).

### 3.4. Measurement of Hydroxyl Radical in Oxidized Sample by TBARS

The TBARS assay has been done as an indicator of oxidative stress. From Figure 2(b), it is clear that the modified sample shows more concentration of hydroxyl radical than native sample. The values of TBARS for native LDL and oxidized LDL were recorded to be 0.12 *µ*M/ml and 0.85 *µ*M/ml, respectively. The extinction coefficient used to determine the content of hydroxyl radical was 1.56*∗*10^5^ M^−1^cm^−1^.

### 3.5. Measurement of Hydroxy Methyl Furfural (HMF)

Similarly, HMF that might have formed in the oxidation of LDL was determined by adding 1M of oxalic acid in 1ml of sample. The HMF content in the oxidised LDL was more than native LDL which had almost negligible level of HMF (Table 1). The HMF contents of native and oxidized LDL were 0.65 and 1.77 *µ*M/ml, respectively (Figure 2(b)). The absorbance was taken at 443 nm and the HMF content was determined using molar extinction coefficient 40,000 M^−1^cm^−1^.

### 3.6. Measurement of Carbonyl Content (CC)

Oxidation of lipoproteins logically results in an increase in protein carbonyl contents, a recognized biomarker of oxidative stress. The carbonyl contents of native and Ox-LDL were 5.8 and 23 *µ*mol mg−1 protein, respectively (Figure 2(c)). This corresponds to increase in carbonyl contents in Ox-LDL as compared to native LDL. The carbonyl contents of native and Ox-LDL were determined using 2, 4-dinitrophenylhydrazine. After taking the absorbance at 370 nm, the carbonyl content was determined by using extinction coefficient 22,000 M^−1^cm^−1^.

### 3.7. Estimation of Free Arginine

L-arginine is converted in the body into a chemical called nitric oxide. Nitric oxide causes blood vessels to open wider for improved blood flow. L-arginine also stimulates the release of growth hormone, insulin, and other substances in the body. As arginine residues are often localized in active allosteric and metal binding centres of enzyme, their oxidation may directly affect the cell status and their physiology of the corresponding author organs and tissues. The decrease in free amino acids in oxidised LDL was evaluated using the following formula:

(1) %  decrease  in  free  amino  group=ε  amino  groups  in  native  LDL-  ε  amino  groups  in  Ox-LDLε  amino  groups  in native  LDL

We observed approximately 24.89% decrease in the level of free arginine in the oxidised sample as compared to the native LDL (Table 1).

### 3.8. Binding of Autoantibodies to T2DM, T2DM+CVD, and CVD Patients Sera

**Figure 3:**
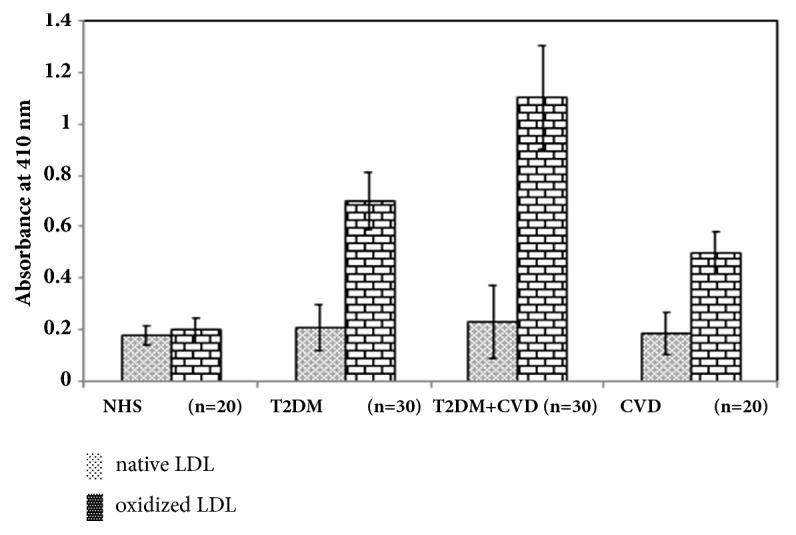
Direct binding ELISA of serum antibodies in T2DM, T2DM+CVD, and CVD against the native and oxidized LDL. Serum from normal human subjects (NHS) served as control. The microtiter plates were coated with the respective antigens (10*μ*g/ml).

The biochemical characteristics of the normal control and patients are given in Table 2. The clinical study was performed to screen out the positive sera samples (sera showing more than double the binding with immunogen) from T2DM, CVD, and T2DM+CVD, respectively, as compare to the normal human sera (NHS). The study comprised of total 100 serum samples amongst which 30 serum samples each are from T2DM+CVD and T2DM, respectively, and 20 serum samples were from CVD group. Twenty control serum samples from age and sex matched individuals were obtained from normal healthy subjects. All sera were diluted to 1:100 in TBS-T and subjected to direct binding ELISA on solid phase separately coated with equal amounts of native LDL and oxidized LDL. The highest binding was observed in T2DM+CVD subjects as compared to T2DM, CVD, and normal human sera (NHS), respectively. Out of 30 sera samples of T2DM+CVD, 15 samples showed enhanced binding when oxidized LDL was used as an antigen. This shows that approximately 50% of the serum shows autoantibodies against oxidized LDL in T2DM having complications of CVD (Figure 3). When the comparison was done with the T2DM and CVD in terms of number of patients' sera showing enhanced binding; eleven T2DM (approx. 36%) and six CVD (30%) patients' sera showed enhanced binding.

## 4. Discussion

Oxidized LDL is cytotoxic to endothelial linings which in turn enhances adhesion of neutrophils that causes endothelial damage and becomes dysfunctional [16]. Furthermore, oxidized LDL adds to the initiation of macrophages, induction of cyclooxygenase expression, and matrix metalloproteinase (MMP-2 and MMP-9) production, which are involved in destruction of fibrous plaque formations [17]. As a result of oxidized LDL uptake by macrophages, foam cells are formed, which further accumulate in the endothelium and contribute to atherosclerotic plaque formation [16]. In the current study, interestingly, the levels of antibodies depended on the type of metabolic disorder a patient has, like type 2 diabetes (T2DM) and CVDs or in combination of both, and the highest antibody levels were observed in T2DM in combination with the CVD patients. This was authenticated by other group of researchers on the fact that there are antibodies to oxidized LDLs in healthy people is well-established, but their levels significantly increase if a patient has cardiovascular disease [17, 18]. Currently, there is no consensus on the role of oxidized LDL antibodies. Some authors believe that the production of antibodies has a protective effect aimed at elimination of atherogenic oxidized LDLs. However, antibodies at increased levels form immune complexes with oxidized LDLs, and cause additional damage to the endothelium [19]. Regardless, most authors believe that antibodies to oxidized LDLs can potentially be atherogenic and their measurement can be used as part of risk assessment [18, 20]. In the present study, we have isolated the LDL macromolecule as discussed above and characterized for its susceptibility to oxidation via the Fenton reaction. The LDL upon oxidation exhibits structural changes as evidenced by the UV-Vis spectroscopy. The increased hyperchromicity was observed in case of oxidised LDL molecule as compared to the native analogue. This might be due to the opening of the LDL structure rendering it to expose the deeply buried aromatic amino acids which upon exposure absorbs more UV light at 280 and increased hyperchromicity.

Various biochemical assays were also performed in order to assess the chemical changes occurring in the LDL molecule. NBT reduction is used to detect concentration of superoxide anion formed in the oxidised LDL system. NBT is a reagent that can absorb superoxide and change its colour to purple or blue (absorbed at 543 nm). In oxidized sample, higher values were observed because of more superoxide concentration than native one. TBARS has been used as an indicator of oxidative stress. Assay of TBARS measures hydroxyl radical present in the sample, using thiobarbituric acid as a reagent. Hydroxyl radical reacts with thiobarbituric acid to give pink colour. In our study, we found approximately eight folds increase in the concentration of hydroxyl radical in oxidised LDL. Hydroxymethylfurfural (HMF) is an organic compound which is enhanced in oxidized sample. HMF can be detected by absorption at 443 nm. Thus, the difference in absorbance, one higher than the other, was used to distinguish between oxidized LDL and native LDL respectively. Carbonyls are widely analyzed as a measure of protein oxidation. The oxidation of proteins results in the production of stable carbonyl groups, which can be used as a measure of oxidative injury. The carbonyl content was measured and it was detected more in oxidized sample as compared to native LDL, and it was confirmed by the higher absorption of Ox-LDL. Arginine is the substrate from which nitric oxide is derived which increases vasodilatation, thus enhancing the easy flow of blood and oxygen through the blood vessel. Due to modification in proteins, the level of L-arginine decreases. In this, a lower free arginine level determined the oxidized LDL as compared to higher free arginine in unoxidized LDL. Furthermore, our work also includes clinical study in which we have used T2DM+CVD patient sera sample. Our study comprised of total 30 sera samples of T2DM+CVD patients where control serum samples from age and sex matched individuals were obtained from 20 normal healthy subjects (Figure 3). High degree of binding was observed in 33% of T2DM+CVD patient's sera towards Ox-LDL, in comparison to its native analogue. NHS showed negligible binding with either antigen.

In the light of above conclusion, human studies on the association of oxidized LDL with different diseases like atherosclerosis or cardiovascular events have been highly conflicting. Some cross-sectional studies suggested a direct association of Ox-LDL antibodies with atherosclerosis in different vascular beds, whereas others found no association with coronary atherosclerotic burden in coronary artery disease patients. Owing to these contradictory results, we focused on T2DM+CVD patients' sera by using Ox-LDL and native LDL as an immunogen. The result reported here clearly shows the immunogenic effect of Ox-LDL against the T2DM+CVD.

## 5. Conclusion

The autoantibodies developed against the oxidised LDL might be used for the early diagnosis and/or prognosis of the diseases associated with it. However, a detailed and pilot study is required for the furtherance in this field. Therefore the need of the hour is to focus on deliverables and the outcomes of similar type of studies.

## Figures and Tables

**Table 1:** Characterization of native and AGE-LDL under identical experimental conditions.

**Parameters**	**Native LDL**	**Ox-LDL**	%** Modifications**
**Absorbance (at 282 nm)**	0.157	0.873	86.25% hyperchromicity
%** increase in superoxide at 525 nm**	0.035	0.085	58.82% increase in superoxide
**HMF content (**µ**mol/ml) at 443 nm**	0.65	1.77	63.27% increase in HMF
**TBARS (**µ**mol/ml) at 532 nm**	0.12	0.85	85.88% increase in TBARS
**Carbonyl content (**µ**mol/mg) at 370 nm**	5.8	23.00	74.78 % increase i
%** decrease in free arginine**			24.89 % decrease

**Table 2:** Biochemical parameters involved in the T2DM and CVD.

**Parameters**	**Normal Human Sera (NHS)**	**T2DM**	**CVD**	**T2DM + CVD**
	**N=20**	**N=30**	**N=20**	**N=30**
**Mean Age, (in years)**	56 ± 5.5	58± 6.4	60± 4.9	64± 5.1
**Fasting glucose (mg/dl)**	90± 12	190± 14	120± 10	180± 14
**Blood pressure (mm Hg)**	114 /82	122/78	134/89	136/90
	± 8/± 5	± 5/± 6	± 9/± 8	± 11/± 9
**TC, mg/dl**	190 ± 15	210± 18	230± 19	260± 21
**TG, mg/dl**	143± 13	198± 17	210± 23	245± 22
**HDL-C, mg/dl**	64± 5.6	53± 6.7	48± 10.1	42± 5.3

## Data Availability

All the data used to support the findings of this study are included within the article.
